# The application of strength and power related field tests in older adults: criteria, current status and a future perspective

**DOI:** 10.1186/s11556-015-0147-6

**Published:** 2015-10-07

**Authors:** G. Ruben H. Regterschot, Tobias Morat, Marjanne Folkersma, Wiebren Zijlstra

**Affiliations:** Center for Human Movement Sciences, University of Groningen, University Medical Center Groningen, Groningen, The Netherlands; Institute of Movement and Sport Gerontology, German Sport University Cologne, Cologne, Germany

**Keywords:** Assessment, Aged, Muscle strength, Muscle power, Sensor

## Abstract

Leg muscle strength (LMS) and leg muscle power (LMP) are determinants of aspects of functional status and important parameters for measuring intervention effects in older adults. Field tests are often used for the evaluation of LMS and LMP in older persons. However, criteria important for the application of strength and power related field tests in older adults have not been systematically taken into account and are not yet fully listed and described in a single publication. Therefore, this paper describes criteria important for the application of strength and power related field tests in older adults. In addition, strength and power related field tests commonly used in older adults are evaluated by using the described criteria. Based on this evaluation, this paper provides a perspective on the further development of field tests. Criteria important for strength and power related field tests are: adequate accuracy, precision, concurrent validity, clinical validity, practical feasibility and pure strength or power outcomes. Commonly used strength and power related field tests do not meet all the aforementioned criteria. Therefore, further development of field tests is necessary. Mobile sensing systems are potentially useful for the evaluation of LMS and LMP in older adults. Mobile sensing systems do not have the limitations of commonly used field tests and provide important additional advantages. In particular, mobile sensing systems offer the opportunity of continuous monitoring during free-movement in the home-environment, thereby reducing the need of standardized assessments by health-care professionals. Future studies should examine the clinical validity of mobile sensing systems and evaluate the application of sensor technology in exercise-based interventions.

## Introduction

Leg muscle strength (LMS) and leg muscle power (LMP) are major determinants of aspects of functional status in older people, such as mobility, activities of daily living and fall risk [1–8]. Therefore, LMS and LMP are considered important parameters for the identification of lower functioning individuals and the evaluation of intervention effects in older adults. Field tests are often used for the evaluation of LMS and LMP in older persons, because field tests are easy-to-use and applicable in clinical settings. Examples of commonly used field tests for the evaluation of LMS and LMP in older adults are hand-held dynamometry (HHD) [9], the Five-Times-Sit-to-Stand Test (FTSST) [10], stair walk tests e.g. [11] and the Short Physical Performance Battery (SPPB) [12].

Strength and power related field tests need to fulfill methodological as well as feasibility criteria. Relevant criteria have been mentioned in different publications e.g. [13, 14]. However, criteria important for the application of strength and power related field tests in older adults have not been systematically taken into account and are not yet fully listed and described in a single publication. As a result, strength and power related field tests are applied in clinical settings without considering the essential methodological and/or practical feasibility criteria. For example, the FTSST has a limited ability to discriminate between higher and lower functioning individuals [10], and stair walk tests may not be feasible because at least half of the older adults between 75–79 years has difficulty with stair walking [15]. Therefore, the present paper describes criteria important for the application of strength and power related field tests in older adults. In addition, this study evaluates strength and power related field tests commonly used in older adults by using the criteria described in the present paper. Based on the results of this evaluation, the present paper also provides a perspective on the further development of strength and power related field tests.

## Criteria for the application of strength and power related field tests in older adults

This section describes criteria important for the application of strength and power related field tests in older adults. The criteria can be categorized into general methodological criteria, criteria related to clinical validity and criteria related to feasibility. Whenever possible, the next sections will be short in addressing specific criteria by referring to further literature which more extensively treats the specific subject.

### General methodological criteria

#### Accuracy

Accuracy is the closeness of agreement between a measured value and the true value or an accepted reference value [16]. For example, a new field test for the measurement of LMP may replace an accepted standard method if the new method shows high accuracy in comparison to an accepted gold standard method. Accuracy is usually quantified as the average difference between target values and measured values.

#### Precision

Precision is the closeness of agreement between repeated measurements [17]. The precision of an instrument that aims to measure a certain physical quantity is calculated by considering the variance of measured values [17]. When regarding the repeated execution of identical measurement procedures, precision consists of repeatability and reproducibility [17]. Repeatability (or test-retest reliability) is the precision when repeated measurements are performed in the same subjects under similar conditions [17, 18]. Reproducibility is the precision when repeated measurements are performed in the same subjects but under different conditions, for example in different environments or with different testers [17].

Adequate repeatability is an important requirement for clinical validity of strength and power related field tests, because field tests should show small variation in outcome during repeated measurements under similar conditions in order to be useful for clinical assessments [19]. Measurement devices are often evaluated on two types of repeatability, namely relative repeatability and absolute repeatability [13, 19]. Relative repeatability is defined as the consistency of the individuals rank in a sample over repeated measurements under similar conditions [19]. Absolute repeatability is defined as the extent to which individual scores vary during repeated measurements under similar conditions [19]. Relative repeatability is frequently evaluated with the intra-class correlation coefficient (ICC) [13, 18–20]. The ICC is often interpreted according to the following criteria: ICC≥0.75 excellent repeatability, 0.40≤ICC<0.75 fair to good repeatability, and ICC<0.40 poor repeatability [21]. The standard error of measurement (SEM) [13, 18, 19] as well as Bland and Altman limits of agreement (LOA) [19, 20, 22] are often used to determine absolute repeatability of a measure. The smaller the SEM and the LOA, the better the absolute repeatability [19]. As to the acceptability of a specific size of SEM or LOA, this depends on specific measurement goals and the size of the differences one wants to measure. Hence, it is not possible to define absolute criteria for SEM and LOA.

#### Concurrent validity

A new measurement instrument demonstrates adequate concurrent validity when the new measurement instrument is associated with the outcomes of a previously validated measure [23]. For example, a new test for the measurement of LMS and LMP has adequate concurrent validity when the new test is associated with the outcomes of a previously validated method for the measurement of LMS and LMP, such as isokinetic dynamometry e.g. [24]. Concurrent validity of a measure is often evaluated using a correlation analysis e.g. [24, 25]. Correlations (r) may be interpreted as follows: little (if any correlation) when 0.00<r≤0.25; weak when 0.26≤r≤0.49; moderate when 0.50≤r≤0.69; strong when 0.70≤r≤0.89; very strong when 0.90≤r≤1.00 [26].

### Criteria related to clinical validity

Discriminative ability and sensitivity to change are important criteria for clinical validity of strength and power related field tests, because they directly relate to diagnosis and evaluation of treatment effects. Both measurement properties are described in more detail below.

#### Discriminative ability

Discriminative ability is a measurement property that can be defined as the ability of a measure to correctly classify subjects into two different groups when true group belonging is known [13]. Adequate discriminative ability is important for clinical validity, because clinical measures should be able to discriminate between subjects with and without a certain condition [13]. For application in clinical settings strength and power related field tests should be able to discriminate between individuals with adequate strength and power, and individuals with insufficient strength and power. Individuals with insufficient LMS and LMP may be selected for participation in an exercise intervention aimed at improving LMS and LMP.

**Fig. 1:**
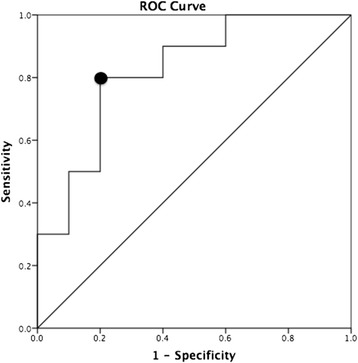
Example of an ROC curve based on fictitious data from a leg power field test. The area under the ROC curve (AUC) is 0.82, indicating a moderately accurate discriminative ability of the field test. The optimal cut-off point is marked with a black circle. The sensitivity and specificity corresponding to the optimal cut-off point are respectively 0.80 and 0.80

Discriminative ability is often evaluated by using the Receiver Operating Characteristic (ROC) curve [13, 27]. ROC curves show the discriminative ability for different cut-off values of the measure (see fictitious data in Fig. 1). The y-axis shows the percentage of subjects with a certain condition correctly classified by the measure as having this condition (sensitivity) and the x-axis shows the percentage of subjects wrongly classified as having the condition (1-specificity) [27]. The discriminative ability of a test is often evaluated by calculating the area under the ROC curve (AUC). The larger the AUC, the higher the discriminative ability of a test. The discriminative ability of a test is considered: non-informative when AUC=0.5, insufficiently accurate when 0.5<AUC≤0.7, moderately accurate when 0.7<AUC≤0.9, highly accurate when 0.9<AUC<1, and perfect when AUC=1 [27].

#### Sensitivity to change

Sensitivity to change is a measurement property that can be defined as the ability of a measure to detect a change over time [13]. In order to be useful in clinical settings, strength and power related field tests should be able to detect changes over time. An adequate sensitivity to change of strength and power related field tests is essential for the evaluation of intervention effects. The change as the result of an intervention should be determined with validated measures before the change can be used to determine the sensitivity to change of a new measure. A high sensitivity to change is the result of a large mean change and a small standard deviation (SD) of change. Sensitivity to change is often evaluated using the standardized response mean (SRM) [13, 28]. The SRM is an effect size measure and is calculated as: SRM = mean change/SD of change [13]. The SRM is considered small when 0.20≤SRM<0.50, moderate when 0.50≤SRM<0.80 and large when SRM≥0.80 [29].

### Outcome measure

According to Horlings et al. (2008) strength measures can be divided into two groups: direct and indirect (surrogate) strength measures [4]. Direct strength measures are those that provide pure strength outcomes [4]. An example of a direct strength measure is HHD. Indirect strength measures are those that do not provide pure strength outcomes. Indirect measures evaluate strength by testing aspects of functional performance [4]. An example of an indirect strength measure is the FTSST [10]. The FTSST provides time duration in seconds as outcome measure and not pure strength or power outcomes in, for example, Newton (N) or Watt (W). Indirect measures of strength test not only muscle strength, but also other aspects, such as coordination [4]. Therefore, if strength or power is an important outcome measure of the study it is recommended to use direct measures of strength or power instead of indirect measures [4].

### Criteria related to practical feasibility

Field tests for the evaluation of LMS and LMP in older adults are developed for use in environments outside the laboratory, such as clinical environments, home settings or community settings. Therefore, strength and power related field tests should be portable, lightweight and executable in a home environment [14, 30]. However, also other factors should be taken into account when considering the practical feasibility of strength and power related field tests: the duration of the measurement, the amount of space required for the measurement, simplicity and acceptability of the test since older adults should be able to perform the test, safety as well as the risk on muscle soreness and injuries, ease of data acquisition and data analysis since clinicians without extensive experience with laboratory devices should be able to administer the test, costs associated with the test and specificity of testing since the movement pattern, contraction type (eccentric, concentric or isometric) and contraction velocity of leg muscles during daily life activities should be transferred to field tests in order to test as specific as possible [14, 30].

## Evaluation of strength and power related field tests

In this section we will evaluate strength and power related field tests commonly used in older adults. First we will summarize findings of the few available studies that evaluated strength and power related field tests commonly used in older adults on aspects of repeatability, validity and practical feasibility. Subsequently we will evaluate field tests considered valid and feasible by these studies using the criteria described in the previous section of the present paper.

A recent review study by Mijnarends et al. (2013) evaluated commonly used strength and power related field tests on aspects of repeatability, validity and practical feasibility in older adults [30]. The results of this review study showed that only HHD, the SPPB and gait speed (GS) over a short distance have adequate repeatability, concurrent validity, construct validity and practical feasibility in older adults. In addition, Stark et al. (2011) concluded that HHD is reliable, valid and practical for the measurement of muscle strength in young as well as older adults [9]. Furthermore, Freiberger et al. (2012) showed that the SPPB has adequate repeatability, responsiveness and validity in older adults [31]. Moreover, Rydwik et al. (2012) concluded that habitual GS is reliable and valid in older adults, however, this study also revealed that the responsiveness of GS is unclear [32]. Together these review studies indicate that HHD, SPPB and GS tests are reliable, valid and feasible in older adults.

However, a critical reflection on HHD, SPPB and GS tests is necessary since these tests have important limitations when the criteria described in the previous section of the present paper are taken into account. First, as already noted, the responsiveness of GS tests is undetermined [32]. For this reason, the clinical validity of GS tests remains unclear and needs further investigation. Second, the SPPB and GS tests do not provide pure strength or power outcome measures. This is a limitation of the SPPB and GS tests since field tests providing pure strength or power outcome measures are recommended, in particular when strength and power are important outcome measures of a study [4]. Third, repeatability of LMS assessments with HHD is inadequate when individuals produce high muscle forces [33]. When an individual produces high muscle forces it is difficult for the therapist to keep the hand-held dynamometer in position during the assessment, thereby reducing the repeatability of the measurement [33, 34]. Fourth, practical feasibility of the SPPB and GS tests is limited, because both tests require a relatively large amount of space, which may be problematic in home settings [31]. Furthermore, the performance of the SPPB requires much time (10-15 minutes) [12] compared to the performance of other field tests (e.g. HHD), which further reduces the practical feasibility of the SPPB. Hence, HHD, SPPB and GS tests have serious limitations, which indicate the need for the development of alternative field tests for the evaluation of LMS and LMP in older adults.

## Future perspective and remaining challenges

The preceding section demonstrated the need for the further development of field tests for the evaluation of LMS and LMP in older adults. In the present section we will provide a perspective on the further development of strength and power related field tests and we will identify remaining challenges.

**Fig. 2:**
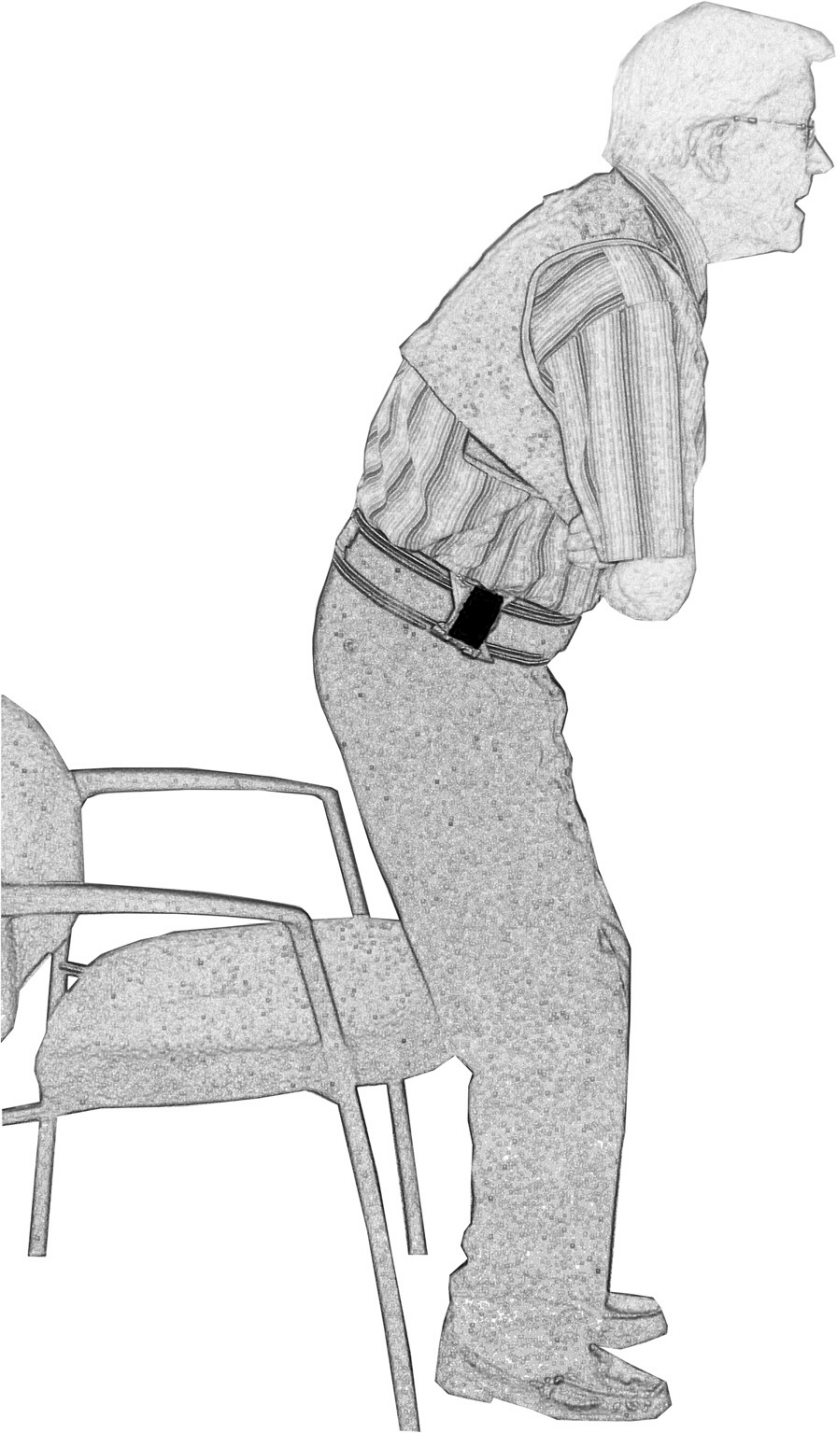
A motion sensor can be used to estimate power during the sit-to-stand movement. The small black box on the right side of the hip represents a body-fixed motion sensor. The vertical acceleration signal measured with the motion sensor can be used to estimate the vertical peak power of the body’s center of mass during the sit-to-stand transfer [40]

With recent developments in technology, mobile sensing systems have become available for the measurement of motor functioning. For example, methods based on body-fixed motion sensors have been developed to assess aspects of mobility in older people e.g. [35, 36]. In addition, gaming systems (e.g. Xbox Kinect, Nintendo Wii) are currently available for the measurement and training of motor functioning in older adults [37]. Moreover, mobile sensing systems have been developed for the measurement of LMS and LMP in older adults. For example, body-fixed motion sensors have been applied to estimate leg power during the sit-to-stand (STS) movement in young and older adults [38–40]. For the estimation of power during STS body-fixed motion sensors have been attached to the right side of the hip and the chest (see Fig. 2) [38–40]. The vertical acceleration signal measured with the body-fixed motion sensors can be used to estimate the vertical peak power of the body’s center of mass during the STS movement [40]. In addition, methods based on force plates are currently available for the assessment of power during the chair rise transfer [41–44]. Force plate methods use the vertical ground reaction force measured with force plates beneath the feet of a person to estimate the vertical power during the STS movement [41–44].

Mobile sensing systems for the measurement of LMS and LMP do not have the limitations associated with commonly used strength and power related field tests. While the SPPB and GS tests do not provide pure strength or power outcomes, mobile sensing systems with pure strength and power outcome measures are already available. For example, a sensor-based method for the evaluation of sit-to-stand performance provides pure power outcomes with adequate concurrent validity [40]. In addition, mobile sensing systems have been developed as a solution for the inadequate repeatability of HHD when high muscle forces are generated. For example, studies integrated force sensors in a fixed station for the assessment of isometric quadriceps strength [34, 45]. By using a fixed station the quadriceps force measurement does not depend on the strength of the therapist. Studies showed that a force sensor integrated in a fixed station provides reliable assessments of quadriceps strength in young adults as well as older adults [34, Douma et al. (submitted)]. Furthermore, in contrast to commonly used strength and power related field tests (such as the SPPB and GS tests) mobile sensing systems for the measurement of LMS and LMP have a high practical feasibility. For example, sensor-based assessments of leg power production during sit-to-stand movements can easily be performed in home settings and take only a few minutes [40, 46]. Furthermore, body-fixed sensor systems are highly portable and lightweight [47].

Mobile sensing systems not only solve the issues associated with commonly used strength and power related field tests, but also offer important additional advantages. In particular, specific mobile sensing systems provide the possibility of assessments in the home environment on a day-to-day or week-to-week basis without a clinician being physically present. For this reason, the clinical relevance of mobile sensing systems can be enormous. This is in particular true for systems based on body-fixed motion sensors. Body-fixed motion sensors can be worn during free-movement in the home environment and are therefore ideal for the continuous monitoring of LMS and LMP in older adults [35, 36, 40, 46, 48]. Continuous monitoring with body-fixed motion sensors during daily life activities has the potential to provide early indications of functional decline and intervention effects. Outcomes of sensor-based assessments can be used by older adults for self-monitoring and by health-care professionals to individualize exercise programs through remote feedback [48]. As a result of this, the performance of standardized assessments by health-care professionals may become less needed. Health-care professionals may only perform a detailed examination of functioning when a functional decline has been detected based on daily life monitoring with sensor technology.

However, the clinical relevance of sensor-based technology for the evaluation of strength and power has not yet been fully demonstrated. With a few exceptions studies report on technical validity and feasibility of sensor technology for the measurement of motor functioning (with the majority of studies showing adequate technical validity and feasibility), but not on the clinical validity of sensor technology [35, 49]. Therefore, future studies should focus on investigating the clinical validity of sensor-based technology in older persons. Another remaining challenge is the development of clinical applications that are commercially available [35]. This requires cooperation of clinicians and industry [35]. In addition, a major challenge is the integration of sensor-based technology in home-based exercise programs e.g. [48]. For this purpose the technology is already available, however, feasibility, adherence and effectiveness of exercise programs driven by sensor-based technology and remote feedback (for example via a tablet PC) are largely unknown and will be addressed in future studies [48].

## Conclusions

The present paper described criteria important for the application of strength and power related field tests in older adults. Based on these criteria, we demonstrated that strength and power related field tests commonly used in older adults each have their very specific limitations. Mobile sensing systems for the evaluation of LMS and LMP solve the issues associated with commonly used field tests and provide important additional advantages. In particular, mobile sensing systems offer the opportunity of continuous monitoring during free-movement in the home-environment, thereby limiting the need for standardized assessments by health-care professionals. Future research should investigate the clinical validity of mobile sensing systems and evaluate the application of sensor technology in exercise-based programs.
